# Supervised learning-based tagSNP selection for genome-wide disease classifications

**DOI:** 10.1186/1471-2164-9-S1-S6

**Published:** 2008-03-20

**Authors:** Qingzhong Liu, Jack Yang, Zhongxue Chen, Mary Qu Yang, Andrew H Sung, Xudong Huang

**Affiliations:** 1 Department of Computer Science, New Mexico Institute of Mining and Technology, Socorro, NM 87801, USA; 2 Institute for Complex Additive Systems Analysis, New Mexico Institute of Mining and Technology, Socorro, NM 87801, USA; 3 Department of Radiology, Brigham and Women's Hospital and Harvard Medical School, Boston, MA 02120, USA; 4 The Children's Hospital of Philadelphia, Philadelphia, PA 19104, USA; 5 National Human Genome Research Institute, National Institutes of Health (NIH), U.S. Department of Health and Human Services, USA; 6 Oak Ridge Institute for Science and Education, Oak Ridge National Laboratory, U.S. Department of Energy, USA

## Abstract

**Background:**

Comprehensive evaluation of common genetic variations through association of single nucleotide polymorphisms (SNPs) with complex human diseases on the genome-wide scale is an active area in human genome research. One of the fundamental questions in a SNP-disease association study is to find an optimal subset of SNPs with predicting power for disease status. To find that subset while reducing study burden in terms of time and costs, one can potentially reconcile information redundancy from associations between SNP markers.

**Results:**

We have developed a feature selection method named Supervised Recursive Feature Addition (SRFA). This method combines supervised learning and statistical measures for the chosen candidate features/SNPs to reconcile the redundancy information and, in doing so, improve the classification performance in association studies. Additionally, we have proposed a Support Vector based Recursive Feature Addition (SVRFA) scheme in SNP-disease association analysis.

**Conclusions:**

We have proposed using SRFA with different statistical learning classifiers and SVRFA for both SNP selection and disease classification and then applying them to two complex disease data sets. In general, our approaches outperform the well-known feature selection method of Support Vector Machine Recursive Feature Elimination and logic regression-based SNP selection for disease classification in genetic association studies. Our study further indicates that both genetic and environmental variables should be taken into account when doing disease predictions and classifications for the most complex human diseases that have gene-environment interactions.

## Background

Correlating DNA sequence variations with phenotypic differences has challenged biomedical research community for decades. Substantial efforts have been made to identify all common genetic variations in humans, including single nucleotide polymorphisms (SNPs), deletions and insertions [1]. The International HapMap Project has collected genotypes of millions of SNPs from populations with ancestry from Africa, Asia and Europe and made this information freely available in the public domain [2-4]. Millions of SNPs have been identified so far, yet, how to best use this information is not always clear. Due to the relatively low power of each SNP amidst the huge number of total SNPs, most researchers are unable to perform a whole genome-wide association study directly based on the genotypes or allele frequencies of individual markers. Nonetheless, a great need exists to develop, both conceptually and computationally, robust algorithms and advanced analytical methods for characterizing genetic variations that are non-redundant. Through this characterization, one can then identify the target SNPs that are most likely to affect the phenotypes and ultimately contribute to disease pathogenesis.

To date the efficiency of searching for optimal set of SNPs has not been efficient. To counter this trend, we propose reconciling information redundancy from associations between SNP markers. This method not only successfully identifies the approximate optimal set of SNPs but also potentially reduces the burden involved with genetic association studies such as time and cost [5].

One primary cause for the lack of success in searching for optimal sets of SNPs is that the high dimensionality and highly correlated features of SNPs hinder the power of identifying small to moderate genetic effects connectable to complex diseases. The need to incorporate covariates of other environmental risk factors as effect modifiers or confounders further worsens “the curse of dimensionality problem” in mapping genes associated with complex diseases [6].

How do we evaluate the searching for optimal SNPs? It must be predetermined prior to searching how SNPs are needed to provide enough predicting power of disease status. This is not a new question; it comes out of the overall recent statistical and computational endeavors that focus on feature selection from massive and highly dimensional genomic data. Specifically, in genome-wide disease association studies, various models and algorithms have been proposed for selecting an optimal subset of SNPs [7-13]. Linkage Disequilibrium-based methods for selecting a maximally informative set of SNPs for disease association analyses have been developed first [14-18]. Zhang and Jin [19] introduced a tagSNPs criterion based on pair-wise Linkage Disequilibrium (LD) and haplotype *r*^2^ measure for case control association studies. Anderson and Novembre [20] and Mannila *et al*. [21] proposed finding haplotype block boundaries using minimum description length. The method presented by Beckmann *et al*. [22] showcases the flexibility of Mantel statistics using haplotype sharing. This method was employed to correlate temporal and spatial distributions of cancer in a generalized regression approach for SNP selections and disease gene mapping. He and Zelikovsky [23] proposed tagSNPs for unphased genotypes based on multiple linear regressions. Other test statistic approaches such as scan statistics by Levin *et al*. [24]; score statistics by Schaid *et al*. [25], and weighted-average statistics [26] were proposed for disease gene mapping in case-control studies and for SNP selection in genetic association studies. By using spliced expressed sequence tags, Yang *et al*. investigated the connection between “bidirectional gene pair” and cancer [35].

Recently, Schwender and Ickstadt [27] demonstrated logic regression [28] based identification of SNP interactions for the disease status in a case-control study and proposed two measures for quantifying the importance of feature interactions and classifications. In comparison with some well-known classification methods such as CART [29], Random Forests [30] and other regression procedures [17], logic regression has been claimed to perform better when applied to SNP data [27].

In this paper, we developed a feature selection method named Supervised Recursive Feature Addition (SRFA). It not only allows for the selection of genomic information but helps to identify the optimal subset of SNPs necessary for finding the variations associated with disease. This method combines supervised learning and statistical measures for the chosen candidate SNPs and/or environmental variables to reconcile redundancy information for improving the classification and prediction performance. We implemented SRFA with different statistical learning classifiers for both SNP selection and disease classification and then compared their performances with popular classification models, such as logic regression and Support Vector Machine Recursive Feature Elimination (SVMRFE). Additionally, we proposed a Support Vector based Recursive Feature Addition (SVRFA) scheme for SNP-disease association analysis. To evaluate and to demonstrate the proposed methods, we applied them to two complex SNP-disease data sets, the Myocardial Infarction Case & Control (MICC) data set and a subset of The North American Rheumatoid Arthritis Consortium (NARAC) data, for both SNP selection and disease classification.

## Results

**Figure 1:**
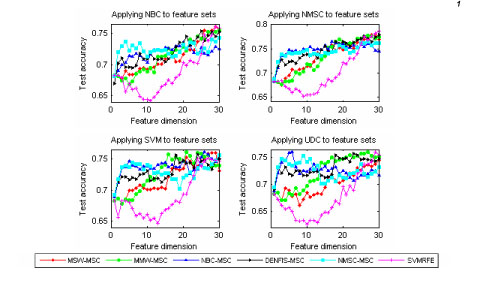
Testing accuracies of NBC, NMSC, SVM, and UDC for the MICC data set. The legend marks the different feature selection methods.

Fig. 1 displays the testing accuracies of NBC, NMSC, SVM, and UDC in the analysis of the MICC data set. The legend marks the different feature selections. Fig. 1 shows that, with the use of the four learning classifiers, both SRFA and SVRFA (including MSW-MSC, MMW-MSC, NBC-MSC, NMSC-MSC, and DENFIS-MSC) outperform the well-known feature selection method SVMRFE. The SRFA methods, NBC-MSC and NMSC-MSC, are better than others including SVRFA. Especially under the low feature dimension, the advantage of SRFA is noticeable. Regarding the classification performances of different learning classifiers, on average, NBC, NMSC, and SVM performed better than UDC.

**Figure 2:**
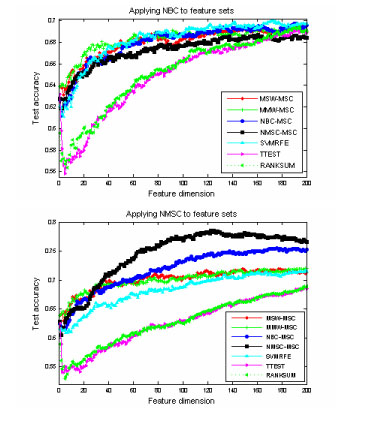
Testing accuracies of NBC and NMSC for the NARAC CHR18SNP data set. The legend marks the different feature selection methods.

Fig. 2 shows the average testing accuracies on the NARAC CHR18SNP case/control data from feature dimension 1 to 200, by applying learning classifiers NBC and NMSC to the following feature selections: MSW-MSC, MMW-MSC, NBC-MSC, NMSC-MSC, SVMRFE, TTEST, and nonparametric RANKSUM. Regarding the testing accuracy, SRFA, SVRFA, and SVMRFE outperform TTEST and RANKSUM. In addition to feature selection, learning classifier is important to the testing performance. MSC combined with RFA helps to improve the classification accuracy. The best testing accuracy is obtained by applying NMSC to the SRFA feature selection, NMSC-MSC. In our view, the weakness of TTEST and RANKSUM is that selection ignores the redundancy and interaction among the SNPs. By contrast, the other approaches may detect the epistatic effects (gene-gene interactions). The detection of higher dimensions of many epistatic effects requires even more complex models.

Overall, regarding the testing accuracies, the feature selection method NMSC-MSC performed the best, followed by NBC-MSC, MMW-MSC, MSW-MSC and SVMRFE; TTEST and RANKSUM performed the worst. Comparing NBC to NMSC, the performance of NMSC is, on average, superior to NBC. Figs. 1 and 2 also show that classification techniques are strictly paired up with feature selections. The performance of NMSC-MSC was not improved by the use of NBC, but with the use of NMSC, the feature selection method NMSC-MSC performed the best.

Tables 1 and 2 list the testing accuracies and the standard errors associated with the highest training accuracies for given classifiers (NMSC, NBC, SVM, UDC) under different feature selections (two SVRFA: MSW-MSC, MMW-MSC; three SRFA: NBC-MSC, NMSC-MSC, DENFIS-MSC; three popular approaches: SVMRFE, Logistic-Wald-t, LOGICFS) for the MICC data set and NARAC CHR18SNP, respectively. In Table 1, the testing accuracies of LOGICFS were obtained from the 31 SNPs in the MICC data set without environmental variables. Although the MICC data set integrates SNPs with environmental variables, due the limit of the number of the features, the differences between the accuracy levels of the tests were not noticeable, although one SVRFA (MMW-MSC) got the best result with the use of NMSC. Table 2 shows that supervised learning-based feature selection NMSC-MSC with the use of NMSC outperforms other combinations, followed by NBC-MSC with the use of NMSC. Support vector based feature selections are superior to LOGICFS, and LOGICFS is better than parametric and non-parametric based feature selections. Regarding support vector based feature selection, on average, MMW-MSC outperformed MSW-MSC and SVMRFE.

**Table 1:** Testing accuracies associated with the highest training accuracies under different feature selections for the MICC data set.

Feature Selection	Testing accuracy (mean value ± standard deviation, %)			
	NMSC	NBC	SVM	UDC
MSW-MSC	76.0 ± 3.4	75.1 ± 3.0	73.1 ± 4.5	73.6 ± 2.9
**MMW-MSC**	**77.4 ± 2.9**	75.9 ± 3.0	74.4 ± 2.3	74.8 ± 4.6
NBC-MSC	75.1 ± 3.1	73.2 ± 2.4	74.2 ± 4.1	75.2 ± 2.6
NMSC-MSC	75.0 ± 4.5	75.0 ± 2.9	74.0 ± 3.7	72.7 ± 3.9
**DENFIS-MSC**	76.9± 3.2	74.2 ± 3.4	**74.9 ± 4.4**	75.6 ± 2.8
SVMRFE	77.0 ± 4.2	73.9 ± 2.7	73.1 ± 4.0	74.4 ± 3.2
T-TEST	75.6 ± 2.6	**76.4 ± 3.0**	74.5 ± 3.1	75.9 ± 3.6
LOGICFS	54.4±1.5			

**Table 2:** Testing accuracies associated with the highest training accuracies under different feature selections for the NARAC CHR18SNP data set.

Feature Selection	Testing accuracy (mean value ± standard deviation, %)	
	**NMSC**	NBC
MSW-MSC	71.3 ± 0.7	68.5 ± 0.7
**MMW-MSC**	71.4 ± 0.4	**69.3 ± 0.3**
NBC-MSC	74.3 ± 0.6	68.3 ± 0.7
**NMSC-MSC**	**77.7 ± 0.7**	67.7 ± 0.3
SVMRFE	67.8 ± 0.8	68.3 ± 0.8
T-TEST	65.4 ± 0.5	66.1 ± 0.8
LOGICFS	67.1 ± 2.1	

## Discussion

Since it is still too expensive to genotype all available SNPs across the human genome, we need advanced approaches to mine the minimum SNPs with the highest prediction accuracy for complex diseases. Our method of exploiting information redundancy from associations among SNP markers provides an efficient and relatively inexpensive method of searching for the optimal or approximate optimal subset of SNPs in genetic association studies. In this paper we specifically propose supervised learning-based strategies, SRFA and SVRFA, to reconcile the redundancy in the highly correlated SNP data to identify the subset of SNPs that enables the most efficient classification of individuals at risk for disease. We evaluated SRA and SVRFA against some popular methods for SNP-disease association studies, and were able to evidence the improvement made by our proposed methods.

Compared with the well-known feature selection methods SVMRFE and LOGICFS, our methods evidenced a higher testing accuracy. When SRFA is associated with two learning classifiers, we have two feature selection methods, NMSC-MSC and NBC-MSC. On average, NMSC-MSC performed better. Among the support vector based feature selection methods, MSW-MSC, MMW-MSC, and SVMRFE, in general, MMW-MSC is the best performer. In comparison SRFA with SVRFA, SRFA performed better than the latter. Our study shows that supervised-learning based MSC feature selection not only reduces the redundancy, but also improves the classification accuracy.

An important factor in the evaluation of testing accuracy worth expounding upon is the training model. In our experiments, training with the use of DENFIS and other neural network classifiers always achieved high training accuracy but the testing accuracy was comparatively not good and over-fitting often happened. Since complex evolutionary learning and classification models, such as DENFIS, almost always require large sample size to elicit their effects, the over-fitting problem is probably related to the relatively small sample sizes. While the complexity of the model increases to achieve higher training accuracy, the requirement for more training samples also increases. If the sample size is not large enough, the relation and model mined from the training samples are not suitable for testing and, as a result, over-fitting happens. This is the reason that complex models fit training samples but not necessarily testing samples very well.

Another point worthy of mentioning is that the learning classifier and feature selection are strictly paired. For instance, NMSC-MSC with the use of NMSC performed the best in the experiments on NARAC CHR18SNP, but NMSC-MSC with the use of NBC was not as good.

The issue of environmental variables also requires discussion. For the MICC data set, with the inclusion of environmental variables, we greatly improved prediction and classification performances. Without the environmental variables, LOGICFS only achieved a 54.4%+/-1.5% correct classification rate. Also, SRFA provided a low (<60%) correct classification rate on the testing data when only using the SNPs, but a higher (>73%) correct classification rate after including the environmental variables as well. These results confirm that complex diseases usually involve both genetic factors and environmental cues. Therefore, both genetic and environmental variables should be taken into account when doing disease predictions and classifications for the most complex human diseases that have gene-environment interactions.

In our experiments, when SVM was applied to the feature sets extracted from the NARAC CHR18SNP genotype data, the classification performance was pretty poor. However, SVM worked well on the feature sets extracted from the MICC data. In our view, the difference might be caused by the following two reasons. First, NARAC CHR18SNP consists of categorical SNP data only, while the MICC data set consists of many environmental variables of which most follow continuous distributions and have major impact on the classification. Second, it might be caused by the failure of optimizing the parameters for the SVM in testing NARAC CHR 18SNP.

Our study shows that, with the use of our methods, even small SNPs and/or environmental variables can obtain good predictive capacity. In the analyses of MICC data, it was evident that, after applying our method with 3-5 variables, we can achieve up to 75% classification accuracy after applying our methods (Fig. 1). On the other hand, SVMRFE needed 20-30 variables in achieving the similar accuracy. In analyses of the NARAC CHR18SNP data set, the advantage of our method is also noticeable (Fig. 2). Experimental results imply that the classification accuracy can be improved and the cost of genotyping can be reduced with the use of our algorithms.

## Conclusions

We proposed SRFA with different statistical learning classifiers and SVRFA for both SNP selections and disease classifications, and then applied them to two complex disease data sets. In general, our approaches outperform the well-known feature selection method of Support Vector Machine Recursive Feature Elimination and logic regression based SNP selection for disease classification involved in genetic association studies. Our study further indicates that both genetic and environmental variables should be taken into account when doing disease predictions and classifications for the most complex human diseases that have gene-environment interactions.

## Materials and methods

### Materials

*Application 1*: Genes and the environment are links between important health conditions: Periodontal Disease (PD) and Cardiovascular Disease (CVD). Cardiovascular disease is the number one cause of death and disability in the Western world. Almost 1 million Americans die of CVD each year, which accounts for 42% of all US deaths. Numerous clinical and epidemiological studies have shown a consistent association between PD and CVD [36], and the link between these two diseases may be the result of common environmental exposures and potential genes that may regulate the individual response to these exposures. The identification of SNPs that influence the risk of diseases through interactions with other SNPs and environmental factors remains a statistical and computational challenge.

The studied Myocardial Infarction Case & Control (MICC) data set is a result of a population based study. The sample included residents of Erie and Niagara counties in New York State, and all were in age group 35 to 69 years. There were 614 white male patients with Myocardial Infarction matched with 614 control males (without CVD) by age (+/- 5 year) and smoking habits; 206 white pre- and post-menopausal females with MI matched with 412 control females (without CVD) by age (+/- 5 year), menopausal status, years since menopause (+/- 2 year), and smoking habits. Diabetics were excluded. The features in the data set included 29 environmental variables, such as two protein variables (ACHMN and CALMEA), which were known to be related to periodontal disease, and smoking status, menopausal status, blood pressure, blood cholesterol, body mass index, drinking status, *etc*. Selection of genetic variables was based on the well-known Seattle web site () by using the candidate approach that included 31 SNPs. This study evaluates the SNP-environment and variable-disease associations especially the effects of SNPs and environmental variables to disease. The original MICC data set contained some missing values. In our experiments, we filtered out the missing values and their associated observations.

*Application 2:* Rheumatoid arthritis (RA) is an autoimmune disease that causes chronic inflammation of joints, tissues around joints, or other organs in body. RA affects more than two million people in the United States. Women account for 70% of patients with RA. While women are two to three times more likely to get RA, men who have RA tend to have more severe symptoms. It afflicts people of all races equally. Onset usually occurs between 30 and 50 years old. Data for this analysis was provided as part of Genetics Analysis Workshop (GAW) 15. The North American Rheumatoid Arthritis Consortium (NARAC), led by Peter Gregersen, has provided microsatellite and SNP scans, quantitative phenotypes, and clinical measures, with additional genotype data provided by Robert Plenge and Ann Begovich. We studied the SNP case-control data named “CHR18SNP.dat” offered by NARAC. In the data file, a dense panel of 2300 SNPs was genotyped by Illumina for an approximately 10 kb region of chromosome 18q. These markers were individually genotyped on 460 cases and 460 controls. Controls were recruited from a New York City population. The objective of this study is to identify SNPs of chromosome 18 that are significantly associated with rheumatoid arthritis. The significant SNPs identified here could be used as a starting point for biologists developing genetic tests that indicate increased risk of developing rheumatoid arthritis.

### Methods

#### Supervised recursive feature addition (SRFA) algorithm for SNP selection

SRFA combines supervised learning and statistical similarity measures among the chosen features and the candidates and is presented as follows:

Step 1: Each individual feature is ranked from the highest classification accuracy to the lowest classification accuracy with the use of a supervised learning classifier.

Step 2: The feature with the highest classification accuracy is chosen as the first feature. If multiple features achieved the same highest classification accuracy, the one with the lowest *p*-value measured by score test-statistics is chosen as the first element. At this point the chosen feature set, *G_1_*, consists of the first feature, *g_1_*, which corresponds to feature dimension one.

Step 3: The (*N+1*)-dimensional feature set, *G_N+1_* = {*g_1_*, *g_2_*, …, *g_N_*, *g_N+1_*}, is produced by adding *g_N+1_* to the current *N*-dimensional feature set *G_N_* = {*g_1_*, *g_2_*,…, *g_N_*}. *g_N+1_* is chosen as follows: Temporarily add each feature *g_i_* (*i ≠ 1, 2, …, N*) outside of *G_N_* to *G_N_*; the classification accuracy of each feature set *G_N_* + {*g_i_*} is then recorded; that *g_c_* which gives the highest classification accuracy is included into the set of candidates, *C*. Generally *C* includes many features, but only one−the feature that is least statistically similar to the already chosen features−will be selected as *g_N+1_* to form the next feature set *G_N+1_*. We call this step Candidate Feature Addition. The goal is to obtain the most informative and least redundant feature set. The statistical similarity measure is based on the Spearman Correlation Coefficient (for categorical features/SNPs) or the Pearson Correlation Coefficient (for continuous environmental variables) between the chosen features *g_n_* (*g_n_* ε *G_N_*, *n* = 1, 2,…, *N*) and the candidate *g_c_* (*g_c_* ε *C, c*= 1, 2 … *m*; *m* is the number of elements in *C*). The Sum of the square of the Correlation (SC) is calculated to measure the similarity and is defined as follows:

$$SC(g_{c})\;=\sum_{n=1}^{N}\;cor^{2}(g_{c,}\;g_{n}),\;n\;=\;1,2\ldots \;N.\;\;$$

where *g_c_* ∈ *C*, *g_n_* ∈ *G_N_*.

The selection of *g_N+1_* follows the qualification that the SC value in (1) is the minimum:

$$\left\{g_{N+1}|\;g_{N+1}\in \;C\cap \;SC(g_{N+1})=\min(SC(g_{c})),g_{c}\in \;C\right\}$$

This strategy is called Minimum SC (MSC).

Step 4: A feature is recursively added to the chosen feature set from steps 1-3 with supervised learning and the similarity measures until classification accuracy stops to increase.

Our SRFA based MSC is denoted as classifier-MSC. For example, if the classifier is Naive Bayes Classifier (NBC), we call the feature selection NBC-MSC.

#### Support vector based recursive feature addition (SVRFA) algorithms

Support Vector Machines (SVMs) [14-16] have been widely applied to pattern classification problems and non-linear regressions. The basic idea of the SVM algorithm is to find an optimal hyper-plane that can maximize the margin between two groups. The vectors that are closest to the optimal hyper-plane are called support vectors. Guyon *et al*. [31] proposed a gene selection utilizing Support Vector Machine methods based on Recursive Feature Elimination (SVMRFE). In addition to gene selection, SVMRFE has been successfully applied to other feature selection and pattern classification issues [37]. Based on the SVMRFE and our SRFA discussed earlier, we propose a Support Vector based lowest weight (or maximum margin width) and the lowest correlation feature addition scheme, called Support Vector based Recursive Feature Addition (SVRFA) described as follows:

1. Train an SVM on each individual feature in the data set to reach an SVM with a weighted vector $g_{j}\in C\text{ | }MW(g_{j})\text{ = min}(MW)$.

2. Rank features according to criterion *c* for feature *i*: *c_i_ =* (*w_i_*)^2^. The features corresponding to the lowest *c* are selected as candidates. The candidate with the highest statistical significance is the first element of the feature set. At this point the chosen feature set, *G_1_*, consists of the first feature, *g_1_*, which corresponds to feature dimension one.

3. The (*N+1*)^*st*^ dimensional feature set, *G_N+1_ =* {*g_1_*, *g_2_*, …, *g_N_*, *g_N+1_*} is produced by adding *g_N+1_* to the *N* dimensional feature set *G_N_* = {*g_1_*, *g_2_*,…,*g_N_*}. The choice of *g_N+1_* is described as follows:

Temporarily add each feature *g_i_* (*i ≠ 1, 2, …, N*) outside of *G_N_* to *G_N_*, train an SVM on feature set *G_N_* + {*g_i_*}, update *c*, and calculate the measures after introducing *g_i_* as follows:

$$SW\text{(}g_{i}\text{) =}\sum_{k=1}^{N+1}c_{k}={\sum_{k=1}^{N+1}w_{k}}^{2}$$

$$MW(g_{i})\text{ =}\max(c_{k})=\max({w_{k}}^{2}),k=1,2...N+1.$$

Here we have two strategies to choose candidates as *g_N+1_*, corresponding to measures *SW* and *MW*, respectively. The candidate set is denoted as *C*. The first strategy is to pick up the feature with the minimum *SW* into *C;* and the second one is based on the minimum *MW*.

$$g_{j}\in C\text{ | }SW(g_{j})\text{ = min}(SW)$$

$$\vec{w}=\sum_{k}\alpha_{k}y_{k}\vec{x_{k}}.$$

Only one feature will be chosen as *g_N+1_*, despite whether set *C* contains multiple candidates or a single one. We chose *g_N+1_* from *C* based on the calculation of SC(*g_j_*), shown in (1), and Minimum SC (MSC) standard, listed in (2).

We call the support vector based Minimum *SW*, calculated in (5), combining with Minimum SC standard, presented in (2) as MSW-MSC. Similarly we call the support vector based Minimum MW in (6) that is combined with Minimum SC in (2) as MMW-MSC. Both MSW-MSC and MMW-MSC are Support Vector based Recursive Feature Addition (SVRFA) algorithms.

#### Implementations and comparison studies

We implemented SRFA with various statistical learning classifiers (with different complexity) proposed in section 2.1. The learning classifiers for feature selections were Naive Bayes Classifier (NBC) [32], Nearest Mean Scaled Classifier (NMSC) [33] and Dynamic Evolving Neuro-Fuzzy Inference System (DENFIS) [34]. We recorded them as NBC-MSC, NMSC-MSC and DENFIS-MSC. Several classifiers including NBC, NMSC, SVM and uncorrelated normal based quadratic Bayes classifier (UDC) [33] were applied to the feature sets selected by the above SRFA in order to compare their performances. Our goals are (i) to evaluate feature selection procedures and find the number of features required for the best classification accuracy; (ii) to evaluate various learning approaches; and (iii) to investigate the redundancy issues in SNP data for improving the classification performance.

We implemented and tested our SVRFA (MSW-MSC and MMW-MSC) methods proposed in section 2.2. For comparison purposes, other popular methods, such as Support Vector Machine Recursive Feature Elimination (SVMRFE), logistic regression based Wald t-test and Logic regression (LOGICFS) for SNP selection and disease classification were compared. In addition, we also applied SVM and other traditional neural network classifiers, such as Levenberg-Marquardt trained feed-forward neural network classifier and back-propagation trained feed-forward neural network classifier [33], for different feature selections to two real data sets. Unfortunately, these learning classifiers didn't work well. Therefore, here we did not list their experimental results.

Cross-validation has been widely used for selecting tuning parameters and optimizing the number of selected genes in the context of building classifiers to avoid over-fitting. We split the data into training and testing samples in each run and built the model based on training samples only and evaluated the performance on the testing samples by using cross-validation. We performed and then tested the accuracy of 20 runs.

## Competing interests

The authors declare that they have no competing interests.

## Authors' contributions

QL performed the study and drafted the manuscript; JY conceived the project and designed the experiments; ZC assisted the study and the manuscript preparation; MQY designed the project and helped to design the algorithms; AHS and XH supervised the study and obtained the supports. All authors have read and approved the final manuscript.
